# The Gap in Digestive Organ Cancers in Inner Mongolia, 2009–2012

**DOI:** 10.5539/gjhs.v7n3p209

**Published:** 2014-11-30

**Authors:** Jie Yang, Agula Bo, Yuan Xia, Hairong Zhang, Xiong Su, Yun Li, Kepeng Xin, Juan Sun

**Affiliations:** 1 Inner Mongolia People’s Hospital, Hohhot, China; 2 Inner Mongolia Medical University, Hohhot, China

**Keywords:** digestive organs, cancer, mortality, potential years of life lost

## Abstract

**Objective:**

The aim of this study was to explore the characteristics of digestive organ cancer mortality and the potential years of life lost in Inner Mongolia, and to provide evidence for the prevention of digestive organ cancers.

**Methods:**

Using data from the Death Registry System from 2009 to 2012, we classified male and female cancer deaths according to the International Classification of Disease (10th revision). The mortality and potential years of life lost were calculated for digestive organ cancers in Inner Mongolia. The average years of life lost was calculated.

**Results:**

Digestive organ cancer mortality in Inner Mongolia was higher in men than in women. The potential years of life lost were also much higher in men than in women. Gallbladder cancer, pancreatic cancer, and colorectal, anus, and anal canal cancer were the most prominent contributors to mortality. Esophageal cancer was the most prominent contributor to potential years of life lost, and was the leading cause of average years of life lost in both sexes.

**Conclusion:**

Liver cancer and stomach cancer mortality and the potential years of life lost to liver and stomach cancer are demonstrably higher in Inner Mongolia. Although esophageal cancer mortality was not the highest of the digestive organ cancers, the average years of life lost to esophageal was the highest for both sexes, and it should therefore be targeted for prevention.

## 1. Introduction

Worldwide, digestive organ cancer deaths represent a substantial public health issue, accounting for approximately 3 million deaths in 2008 (Jemal et al., 2011). In China, cancer is the second leading cause of death, after cerebrovascular diseases, and digestive organ cancer is the leading cause of cancer deaths (Lingzhi & Ping, 2010). Digestive organ cancer is also a public health problem in China and accounts for 55% of all male and 44% of all female cancer deaths (Wanqing et al., 2014). The Chinese society has undergone many changes during recent decades. With the development of society and the economy, the population’s living and diet habits have changed, and combined with an aging population, this has meant that digestive organ cancer is a heavy social and economic burden.

To identify and prioritize causes of premature death, the potential years of life lost (PYLL) is used as an analytical tool in our study. PYLL can be measured for a comprehensive set of conditions. PYLL, as opposed to more traditional mortality measures, highlights premature deaths. These deaths are particularly important from a public health and public policy perspective because they represent preventable loss of life (Carter & Nguyen, 2012).

To the best of our knowledge, no studies have evaluated digestive organ cancer mortality and PYLL in Inner Mongolia. The aim of the present study was to determine the mortality and PYLL associated with digestive organ cancer in Inner Mongolia from 2009 to 2012.

## 2. Materials and Methods

Data on deaths were collected from the Death Registry System (DRS), as previously described (Kepeng et al., 2014). Only data from 2009 to 2012 were used.

The DRS includes information on the primary cause of death, sex, and age. The cause of death was coded according to the International Classification of Disease, 10th revision (ICD-10). In this study, we included deaths that were assigned ICD-10 codes for esophageal cancer (C15), stomach cancer (C16), small intestine cancer (C17), colorectal, anus, and anal canal cancer (C18–C21), liver cancer (C22), gallbladder and unspecified parts of the biliary tract cancer (gallbladder cancer, C23 and C24), pancreatic cancer (C25), and other and ill-defined digestive organ cancers (C26).

Population data from 2009 to 2012 were obtained from the Center for Disease Control and Prevention of Inner Mongolia to calculate cancer mortality. The mortality rates for different digestive organ cancers were calculated. The χ^2^ test was used to examine differences in digestive organ cancer mortality from 2009 to 2012. PYLL, corresponding to the mortality percentages and average years of life lost (AYLL), which equates to the PYLL per death, were calculated. PYLL was calculated using the formula PYLL = Σ (ai × di), where ai is years of lost life for a certain age group, and di is the number of deaths in that particular age group. AYLL = PYLL / Σdi. The different mortality percentages and PYLL were compared. The AYLL for different digestive organ cancers was compared for both sexes.

The statistical significance level was set at P ≤ 0.05 (two-sided). All statistical analyses were performed using SPSS 13.0.

## 3. Results

Between 2009 and 2012, 5371 deaths from digestive organ cancers were recorded in the DRS. The change in mortality during the 4 years was not significant (χ^2^, 4.9; P = 0.176). Mortality and PYLL were calculated using data from all 4 years. Table 1 presents the mortality rates for digestive organ cancers for both sexes. The mortality rate of liver cancer was highest for both men and women (26.1/100,000 and 9.5/100,000, respectively). In men, stomach cancer and esophageal cancer ranked the second and third biggest killers, respectively. The mortality rate for stomach cancer was 22.1/100,000 and for esophageal cancer was 16.8/100,000. Similarly, stomach cancer and colorectal, anus, and anal canal cancers ranked the second and third biggest killers for women, respectively. The mortality rate for stomach cancer was 8.0/100,000 and for colorectal, anus, and anal canal cancer was 5.6/100,000.

**Table 1:** The mortality rates (1/10^5^) for malignant neoplasms of the digestive organs in Inner Mongolia by sex, 2009–2012

Cause of death	Mem	%	Women	%	Total	%
Esophageal cancer	16.79	21.1	2.66	8.7	9.94	17.8
Stomach cancer	22.14	27.9	8.04	26.3	15.31	27.5
Small intestine cancer	0.54	0.7	0.19	0.6	0.37	0.7
Colorectal, anus, and anal canal cancer	7.23	9.1	5.55	18.2	6.41	11.5
Liver and intrahepatic bile duct cancer	26.11	32.9	9.47	31.0	18.05	32.4
Gallbladder cancer	1.09	1.4	1.11	3.6	1.10	2.0
Pancreatic cancer	5.31	6.7	3.39	11.1	4.38	7.9
Other and ill-defined digestive organ cancers	0.24	0.3	0.11	0.4	0.18	0.3
Total	79.46	100	30.51	100	55.75	100

Table 2 presents the PYLL to digestive organ cancers in Inner Mongolia by sex and shows the rank order of the leading causes of PYLL. The leading cause of PYLL for men was liver cancer, followed by stomach cancer and esophageal cancer, while in women, the leading cause of PYLL was stomach cancer, followed by liver cancer and esophageal cancer.

**Table 2:** The potential years of life lost to malignant neoplasms of the digestive organs in Inner Mongolia by sex, 2009-2012

Cause of death	Men	%	Women	%	Total	%
Esophageal cancer	12176	22.5	5253	18.2	18527	21.4
Stomach cancer	14603	27.0	8359	29.0	23994	27.7
Small intestine cancer	352	0.6	232	0.8	601	0.7
Colorectal, anus, and anal canal cancer	4722	8.7	3906	13.5	8736	10.1
Liver and intrahepatic bile duct cancer	18778	34.7	8258	28.6	28457	32.8
Gallbladder cancer	433	0.8	583	2.0	966	1.1
Pancreatic cancer	2939	5.4	2198	7.6	5248	6.0
Other and ill-defined digestive organ cancers	152	0.3	47	0.2	216	0.2
Total	57083	100	29597	100	90102	100

**Figure 1:**
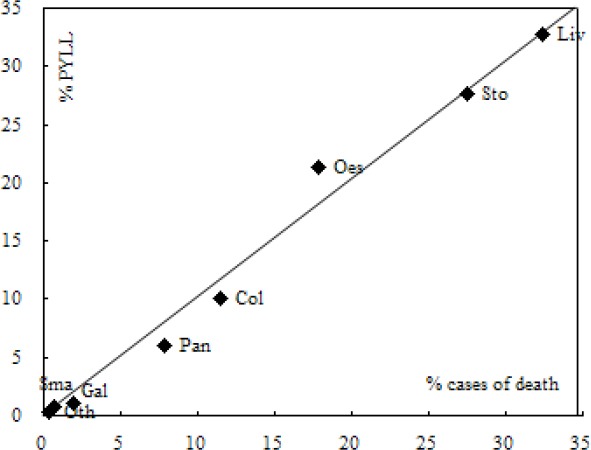
Potential years of life lost (PYLL) versus mortality, Inner Mongolia 2009-2012

Figure 1 presents the proportions of PYLL and corresponding cause-specific mortality rates for the same 5371 deaths. There are evident differences between these 2 approaches of describing cause-specific mortality. Points to the left of the line of equality (y = x) indicate causes that contribute to the numbers of dead more than to the PYLL. Many points lie close to the line of equality. Liver cancer, stomach cancer, small intestine cancer, and other and ill-defined digestive organs cancers are represented by similar percentages of PYLL and mortality. Esophageal cancer is a more prominent contributor to PYLL, while gallbladder cancer, pancreatic cancer, and colorectal, anus, and anal canal cancer largely affect the elderly.

**Figure 2:**
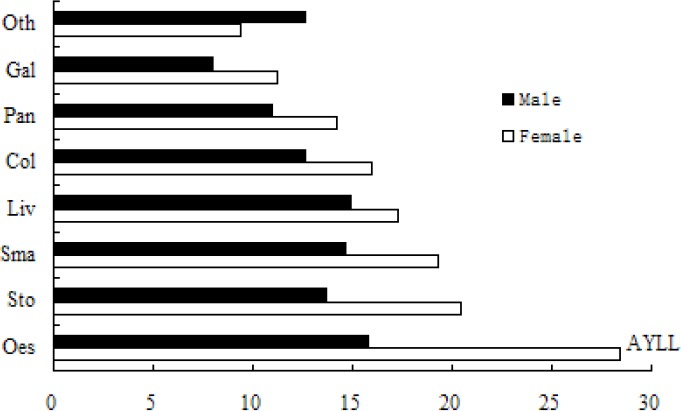
The AYLL by cause of death for males and females, Inner Mongolia 2009-2012

Figure 2 indicates that women experienced higher AYLL than men for all digestive organ cancer causes, except other and ill-defined digestive organ cancers. Esophageal cancer, liver cancer, and small intestine cancer contributed most to the AYLL for men. The leading cause of AYLL was esophageal cancer for women, which was significantly higher than other digestive organ cancers; stomach cancer and small intestine cancer ranked the second and third, respectively, for women.

## 4. Discussion

Cancer is the second leading cause of death, after circulatory system diseases, in Inner Mongolia, and digestive organ cancers are the biggest contributor to cancer related deaths (Ying et al., 2014).

Both mortality and PYLL were higher in men than in women in Inner Mongolia during 2009–2012. Similar findings have been reported elsewhere in China (Qiong et al., 2012; Haiyan, Xiaolan, & Dekun, 2013). Other studies have also identified higher digestive organ cancer mortality rates for men than women in most other countries (Jung et al., 2013). The sex discrepancy (79.5 vs. 30.5 per 100,000) is similar in the Korean population (93.1 vs. 53.9 per 100,000; Jung et al., 2013).

In our study, the PYLL to liver cancer accounted for 35% of all male and 29% of all female digestive organ cancer deaths. Some studies have shown that the burden of liver cancer is most serious in developing countries, especially in China, and accounts for half of deaths worldwide (Ferlay et al., 2010). Similarly, liver cancer accounted for 33% of male and 31% of female mortality in this study. This is consistent with a previous study that indicates that liver cancer is the first most frequent cause of digestive organ cancer death in men (Jemal et al., 2011). Our previous study has also shown that both the PYLL and mortality rates for liver cancer ranked first in digestive organ cancers (Ying et al., 2014). The high liver cancer mortality rate in Inner Mongolia largely reflects the prevalence of chronic hepatitis B and hepatitis C virus infection (Rui, Wenrui, Shaohong, & Guisen, 2009). Our study shows that the mortality rate for liver cancer is more than 3 times higher in men than in women. Alcohol-related cirrhosis and possibly nonalcoholic fatty liver disease are thought to be the cause of high liver cancer rates in men (El-Serag, 2007).

The PYLL to stomach cancer accounted for 27% of all male and 29% of all female digestive organ cancer deaths. The analysis indicated that the loss of life due to stomach cancer deaths was more serious in women than in men. Similarly, the AYLL to stomach cancer was higher in women than in men. However, the mortality rate for stomach cancer was higher in men than women. This is consistent with a previous study (Seoane-Mato et al., 2014). Stomach cancer was the leading cause of digestive organ cancer death in the 1960s (Seoane-Mato et al., 2014). Recently, its mortality has been decreasing (Haga et al., 2013). Even today, stomach cancer mortality remains high in most parts of the world. Epidemiological studies have demonstrated that chronic gastritis, caused by *Helicobacter pylori* is a strong risk factor for stomach cancer (Cover, Krishna, Israel, & Peek, 2003). Our previous studies have also shown that *H. pylori* and NaCl are associated with stomach cancer (Sun et al., 2006). Meat consumption, especially beef and mutton, is significantly higher in Inner Mongolia, and eating meat has been shown to be associated with digestive organ cancer (Kepeng et al., 2014). Stomach cancer and liver cancer have been ranked as the leading digestive organ cancers for PYLL in the latter half of 20th century (Sun et al., 2001). Our current study also shows similar results.

The PYLL to esophageal cancer accounted for 21% of all PYLL and 18% of all deaths from digestive organ cancers. This indicates that esophageal cancer deaths occur at a comparatively young age. The AYLL to esophageal cancer are the highest for all digestive organ cancers in both men and women. Especially for women, the AYLL are far greater than for other digestive organs cancer. Compared to the AYLL, the mortality rate for esophageal cancer is higher in men than in women. This result is consistent with a previous study (Jemal et al., 2011). China has one of the highest esophageal cancer mortality rates around the world, especially North-Central China (Jemal et al., 2011). Recently, although mortality has gradually decreased, mortality remains high in China (Siwei et al., 2012). The esophageal cancer mortality rate of Inner Mongolia is associated with smoking and excessive alcohol consumption (Ju, Xiong, Dan, & Yonghong, 2005). Similarly, a number of other studies have also found that smoking, excessive alcohol consumption, and chronic gastroesophageal reflux disease are risk factors for esophageal cancer death (Kamangar, Chow, Abnet, & Dawsey, 2009).

The characteristics of PYLL and mortality for pancreatic cancer and gallbladder cancer are consistent with colorectal, anus, and anal canal cancer.

Our study indicates that PYLL is a useful indicator to present to policy makers in conjunction with more traditional mortality rates. Although the mortality rate for esophageal cancer was not the highest, the PYLL adds value in demonstrating the effect on the population for each individual death. The PYLL can give a greater priority to younger age groups (Gunnell & Middleton, 2003).

## 5. Conclusions

This article has quantified the differences in mortality and PYLL for digestive organ cancer deaths in Inner Mongolia. Liver cancer and stomach cancer have demonstrably higher mortality rates and account for the most PYLL in Inner Mongolia. Our study has provided important information for policy development for cause-specific prevention strategies in Inner Mongolia.
